# LRRK2 and Protein Aggregation in Parkinson’s Disease: Insights From Animal Models

**DOI:** 10.3389/fnins.2020.00719

**Published:** 2020-07-08

**Authors:** Dylan J. Dues, Darren J. Moore

**Affiliations:** Center for Neurodegenerative Science, Van Andel Institute, Grand Rapids, MI, United States

**Keywords:** Parkinson’s disease, LRRK2, tau, alpha-synuclein, protein aggregation, neurodegeneration, animal models

## Abstract

Mutations in *leucine-rich repeat kinase 2* (*LRRK2*) instigate an autosomal dominant form of Parkinson’s disease (PD). Despite the neuropathological heterogeneity observed in *LRRK2*-PD, accumulating evidence suggests that alpha-synuclein and tau pathology are observed in a vast majority of cases. Intriguingly, the presence of protein aggregates spans both *LRRK2*-PD and idiopathic disease, supportive of a common pathologic mechanism. Thus, it is important to consider how LRRK2 mutations give rise to such pathology, and whether targeting LRRK2 might modify the accumulation, transmission, or toxicity of protein aggregates. Likewise, it is not clear how LRRK2 mutations drive PD pathogenesis, and whether protein aggregates are implicated in LRRK2-dependent neurodegeneration. While animal models have been instrumental in furthering our understanding of a potential interaction between LRRK2 and protein aggregation, the biology is far from clear. We aim to provide a thoughtful overview of the evidence linking LRRK2 to protein aggregation in animal models.

## Introduction

Leucine-rich repeat kinase 2 (LRRK2) is a large multidomain and dual enzymatic protein that exists intracellularly and predominantly as a dimer in its active form (Islam and Moore, 2017). LRRK2 has a number of purported functions, the most well studied of which include roles in the endolysosomal pathway and autophagy (Berwick et al., 2019; Cunningham and Moore, 2020). Most significant, however, is the role that LRRK2 plays in human health. Mutations in *LRRK2* are a common genetic cause of Parkinson’s disease (PD) (Biskup and West, 2008). To date, at least seven missense mutations have been identified as causative in disease. These pathogenic mutations appear to cluster within the Roc-COR tandem (R1441C/G/H, N1437H, and Y1699C) and kinase (G2019S and I2020T) domains, implicating both GTPase and kinase activity in disease pathogenesis (Healy et al., 2008; Islam and Moore, 2017). Additional findings from GWAS have repeatedly linked variation at the *LRRK2* locus as an important risk factor in sporadic PD susceptibility (Nalls et al., 2014, 2019). Thus, LRRK2 represents a pleomorphic risk locus for PD pathogenesis as both a causative and risk-modifying factor.

Familial PD caused by *LRRK2* mutations (*LRRK2*-PD) generally develops as a late-onset autosomal dominant disorder and is thought to be driven by a toxic gain-of-function mechanism (Paisán-Ruıíz et al., 2004; Zimprich et al., 2004). A consensus view holds that elevated kinase activity underlies the pathogenic nature of LRRK2, as all causative mutations appear to enhance kinase activity, though GTPase activity seems to be required as well (Sheng et al., 2012; Steger et al., 2016; Nguyen et al., 2019). Despite *LRRK2* mutations being one of the most common identifiable causes of PD, the vast majority of PD occurs via an unknown etiology. In spite of this, recent evidence suggests that LRRK2 activation (in the absence of mutation) may be present in sporadic PD (Maio et al., 2018). While being clinically indistinguishable from sporadic disease, *LRRK2*-PD frequently harbors neuropathological features synonymous with sporadic PD (Biskup and West, 2008). These features include dopaminergic neurodegeneration in the midbrain along with the appearance of intraneuronal proteinaceous aggregates. These inclusions are generally associated with neurotoxicity, and inclusion burden in cortical and limbic regions is associated with the manifestation of cognitive and neuropsychiatric symptoms, a feature that spans both sporadic disease and *LRRK2*-PD (Aarsland et al., 2005; Braak et al., 2005; Goldwurm et al., 2006; Smith et al., 2019). Thus, persuasive clinical findings infer that mutations in *LRRK2* might influence PD-associated pathology and provoke PD pathogenesis through mechanisms similar to those present in sporadic disease. While the application of LRRK2 pathobiology to idiopathic PD is alluring, it is critical that conserved pathologic features are reconciled with experimental evidence. Accordingly, the contribution of LRRK2 toward protein aggregation is of great interest and likely to be of therapeutic relevance.

A looming question in LRRK2 pathophysiology is whether protein aggregation is a primary or secondary effect of *LRRK2* mutations. Given the variety of neuropathology in *LRRK2*-PD, in many cases being diverse (i.e., co-pathology), it remains to be elucidated whether aggregation is simply resultant to LRRK2-mediated cellular dysfunction or whether pathogenic LRRK2 activity plays a more direct role in driving inclusion formation. Likewise, the requirement of protein aggregate substrates, such as α-synuclein or tau (reviewed in Moussaud et al., 2014), in mediating LRRK2-dependent neurodegeneration is far from clear. Notably, “pure” nigral degeneration in the absence of protein aggregate pathology, though rare, has been observed in *LRRK2*-PD (Gaig et al., 2007; Takanashi et al., 2018). While it is possible that soluble oligomeric protein or other toxic species are still present in these atypical cases, the non-appearance of typical Lewy pathology composed of insoluble fibrillar α-synuclein, or neurofibrillary tangles composed of tau, suggest that inclusion pathology may be a by-product of *LRRK2*-PD pathogenesis rather than a required element. Even when present, it may be the case that the composition of protein aggregates in *LRRK2*-PD is wholly distinct from that of sporadic disease. Toward this concept, examination of α-synuclein derived from the *LRRK2*-PD brain was found to possess a divergent biochemical profile despite a comparable histopathological appearance to sporadic PD (Mamais et al., 2013). Further, the range of pathological findings in the *LRRK2*-PD have been far from consistent. Though accruing post-mortem studies suggest that the common G2019S mutation appears to more faithfully produce typical Lewy pathology, examination of *LRRK2*-PD caused by other mutations has found substantial variation. Divergent pathology has even been observed in *LRRK2*-PD cases between affected members of the same family harboring an identical missense mutation (Zimprich et al., 2004). Thus, while the specific *LRRK2* mutation may have an impact on neuropathological variation, it appears likely that stochastic events may also play a role in the formation of protein aggregates. Though the apparent heterogeneity of *LRRK2*-PD neuropathology might at first suggest that inclusion formation is merely non-specific, accumulating evidence suggest a more selective and subtle impact of LRRK2 in facilitating protein aggregation. Toward addressing some of these questions, past and present genetic studies have granted some insight into the interplay of LRRK2 and the aggregate-prone proteins, α-synuclein and tau.

Common variation at the *SNCA* (encoding α-synuclein) and *MAPT* (encoding tau) loci have been associated with PD risk (Davis et al., 2015). Additionally, variation at these same loci has been implicated in affecting aspects of *LRRK2*-PD, such as the age of disease-onset (Kobayashi et al., 2006; Golub et al., 2009; Gan-Or et al., 2011; Botta-Orfila et al., 2012). Thus, LRRK2, tau, and α-synuclein appear to converge in mysterious and seemingly complex ways in PD pathogenesis. Accordingly, elucidation of the interplay between LRRK2 and PD-associated proteins α-synuclein and tau is highly relevant to the application of LRRK2-targeted therapeutics in suppressing PD-associated neuropathology. Here, we will provide a concise review of current advances in understanding the contribution of LRRK2 toward protein aggregation in human studies and experimental models.

## Protein Aggregation in Human *LRRK2*-PD

Substantial evidence supports a role for protein aggregation in the pathophysiology of PD (Moore et al., 2005). In sporadic disease, the presence of Lewy pathology composed of misfolded α-synuclein protein is a neuropathological hallmark. While predominantly restricted to the brainstem, Lewy pathology is believed to seemingly progress in a caudal to rostral fashion throughout the brain (Braak et al., 2003; Brundin and Melki, 2017). The observation of Lewy pathology in surviving dopaminergic neurons of the substantia nigra has led to the hypothesis that inclusions are ultimately toxic, leading to the progressive loss of dopaminergic innervation of the striatum and associated hypokinetic motor phenotype (Poewe et al., 2017). Further, an abundance of Lewy pathology in cortical and limbic brain regions has been associated with the clinical correlates of dementia and neuropsychiatric disease, respectively (Aarsland et al., 2005; Braak et al., 2005; Smith et al., 2019). Thus, the presence of α-synuclein aggregation appears to drive a heterogenous clinical phenotype by disrupting functional circuits in addition to driving progressive neurodegeneration.

Importantly, protein aggregation encompasses both sporadic and *LRRK2*-PD, suggesting a common pathologic basis of disease. However, whether neurodegeneration in sporadic and *LRRK2*-PD are equally dependent on protein aggregation, and whether all *LRRK2* mutations act through a common pathway is unknown. While progressive degeneration of dopaminergic neurons in the substantia nigra pars compacta remains the primary pathology, a feature that is universal across PD etiology, the nature, extent, and contribution of protein aggregation is far from clear. The majority of post-mortem neuropathological assessments have demonstrated that typical Lewy pathology is a predominant finding in *LRRK2*-PD (Biskup and West, 2008). This is likely due to the overrepresentation of the G2019S mutation in post-mortem studies, as the G2019S mutation is by far the most common *LRRK2* mutation and appears to present with typical Lewy pathology. Pathological examination of *LRRK2*-PD brains harboring the G2019S mutation has identified typical, transitional, and diffuse Lewy pathology, tau and neurofibrillary tangle pathology, TDP-43 aggregates, ubiquitin inclusions, and even “pure” neurodegeneration without overt pathology (Paisán-Ruıíz et al., 2004; Zimprich et al., 2004; Biskup and West, 2008; Wider et al., 2010; Ujiie et al., 2012; Ling et al., 2013). Thus, while α-synuclein aggregation appears to be the most prevalent finding, neuropathology in *LRRK2*-PD is pleomorphic. Aside from the G2019S mutation, a number of other post-mortem studies incorporating less common *LRRK2* mutations have discovered equally diverse pathological findings. Notably, several members of an affected family harboring the R1441C mutation were revealed to exhibit either Lewy pathology, neurofibrillary tangle pathology, or ubiquitin pathology (Zimprich et al., 2004). Ubiquitin inclusions in the absence of Lewy pathology was also observed in two patients with the Y1699C mutation (Zimprich et al., 2004). Intriguingly, no inclusions were detected in a patient with the R1441G mutation (Martí-Massó et al., 2009). Similarly, the I2020T mutation was demonstrated to induce nigral degeneration without significant Lewy pathology, though some cases were later found to harbor extensive brainstem tau pathology (Ujiie et al., 2012; Takanashi et al., 2018).

More recent observations have amplified the significance of tau pathology in *LRRK2*-PD. Indeed, a recent examination of post-mortem *LRRK2*-PD brains further illuminated the prominence of tau aggregation as a common pathologic substrate in *LRRK2*-PD (Henderson et al., 2019c). Though the sample size was limited, a larger proportion of *LRRK2*-PD brains were found to harbor tau pathology relative to α-synuclein pathology (100% vs. ∼64%). While phosphorylated α-synuclein (pSer129+) pathology was abundant in the substantia nigra, amygdala, hippocampus, and cingulate cortex, phosphorylated tau (AT8+) was more prominent in the amygdala, hippocampus, and entorhinal cortex. The regional mapping of each particular protein to a pathologic “territory” is plausibly in reference to a cell or tissue-dependent aspect of LRRK2 pathobiology. Given the preponderance of *LRRK2*-PD cases harboring mixed pathological features, future studies should make note of the regional distribution of pathology in dissecting mechanisms related to LRRK2 and protein aggregation. Returning to the findings of the above study, the midbrain was determined to be largely devoid of tau pathology, suggesting that while tau aggregation may be a typical pathologic feature in *LRRK2*-PD, the application of tau pathology to dopaminergic neuron loss remains ambiguous. The burden of tau pathology was, however, strongly associated with the level of α-synuclein pathology, reminiscent of evidence supporting the potential of tau and α-synuclein cross seeding (Guo et al., 2013). Neither tau nor α-synuclein burden was found to be associated with the level of amyloid-β pathology, further supporting a synergistic relationship between tau and α-synuclein rather than a general disruption in neuronal proteostasis. While a direct interaction between LRRK2 and tau or α-synuclein seems unlikely, it is notable that most regions harboring mixed pathology only rarely exhibited co-pathology within the same cell. It is interesting to hypothesize why some neuronal populations appear to foster one type of protein aggregate, while other populations of neurons another. As well, from an *in vivo* perspective, the requirement of tau or α-synuclein cross seeding in fostering LRRK2-dependent neurodegeneration is fascinating. Toward this concept, examining region-autonomous variation in LRRK2 biology may be a promising endeavor. While human studies have proven invaluable to our collective understanding of protein aggregation in *LRRK2*-PD, much of what is known regarding the mechanistic role of LRRK2 in driving protein aggregation and neuropathology has been garnered from animal models.

## LRRK2 and Protein Aggregation in Animal Models

The identification of pathogenic mutations in the *LRRK2* gene has allowed for the generation of *LRRK2*-PD animal models. These models have provided valuable insight into the biology and pathobiology of LRRK2, though many key questions remain unanswered. While extensive work has been conducted in the development and characterization of these models, here, we will focus specifically on observations of protein aggregation and neuropathology. Toward this aim, experimental findings from traditional genetic, viral vector, and protein-based animal models will be discussed.

### LRRK2 in Transgenic and Knockin Models

Multiple pathogenic substitutions in the R1441 residue of the GTPase domain have been identified in *LRRK2*-PD and are associated with variable neuropathology (Zimprich et al., 2004). Some of the first LRRK2 animal models sought to examine the impact of mutations at this residue in the rodent brain (Table 1), and the development of R1441C knockin mice led to a primary identification of altered dopaminergic neurotransmission. However, the integrity of the nigrostriatal tract in R1441C knockin mice remained relatively unaffected (Tong et al., 2009). Furthermore, biochemical and histological assessment of these mice failed to detect any indications of tau, α-synuclein, or ubiquitin aggregation (Tong et al., 2009). In contrast, a BAC transgenic mouse line expressing human R1441G LRRK2 was found to harbor modest signs of tau pathology. Specifically, elevated protein levels of phosphorylated tau were identified in R1441G BAC mice relative to controls. In addition, neuritic inclusion pathology (AT8+) was observed in the cortex and striatum (Li et al., 2009). While neurodegeneration was not observed in either of these genetic models, subtle alterations in the dopaminergic system were detected, along with a promising implication for involvement of tau.

**TABLE 1:** LRRK2 in transgenic and knockin models.

References	Model	LRRK2 variant(s)	Neurodegeneration	Pathology/Protein aggregation	Additional findings
Tong et al. (2009)	LRRK2 knockin mice	R1441C	Not observed	No protein aggregation was observed, tau and α-synuclein protein levels were not altered	Altered DA neurotransmission
Li et al. (2009)	LRRK2 BAC Tg mice	R1441G, Wild-type	Not observed	R1441G mice harbored elevated protein levels of hyperphosphorylated tau, AT8+; axonal swellings and dystrophic neurites were observed in the dorsal striatum and piriform cortex α-synuclein protein was not assessed	R1441G mice displayed age-dependent motor activity deficits
Lin et al. (2009)	CamKIIα-tTA inducible LRRK2 Tg mice crossed with CamKIIα-tTA inducible α-synuclein A53T Tg mice	G2019S, Wild-type	Both G2019S and wild-type LRRK2 expression was found to exacerbate striatal and cortical neuronal loss	G2019S/A53T bigenic mice were found to have elevated levels of insoluble α-synuclein protein	G2019S/A53T bigenic mice displayed enhanced astrogliosis and microglial activation
	LRRK2 knockout mice crossed with CamKIIα-tTA inducible α-synuclein Tg mice	LRRK2^–/–^	LRRK2 deletion was shown to reduce the extent of striatal and cortical neuronal loss	LRRK2 deletion was found to reduce the level of insoluble α-synuclein protein	LRRK2 knockout mice displayed a reduction in the level of astrogliosis and microglial activation
Melrose et al. (2010)	LRRK2 BAC Tg mice	G2019S, Wild-type	Not observed	G2019S mice displayed mislocalization and increased phosphorylation of tau No abnormalities related to α-synuclein were detected	Altered DA neurotransmission, G2019S mice display anxiety-like behaviors
Li et al. (2010)	LRRK2 BAC Tg mice	G2019S, Wild-type	Not observed	α-synuclein and ubiquitin protein levels were not altered, wild-type mice displayed fewer PHF-1+/CP13+ cells in the dorsal striatum relative to control and G2019S mice	Altered DA neurotransmission
Ramonet et al. (2011)	CMV-PDGFβ-driven LRRK2 Tg mice	G2019S, R1441C, Wild-type	Age-dependent DA neuronal loss was observed in G2019S mice	Abnormal phosphorylated tau and α-synuclein immunostaining was not detected	G2019S mice accumulate autophagic vacuoles
Chen et al. (2012)	CMV-PDGFβ-riven LRRK2 Tg mice	G2019S, Wild-type	Age-dependent DA neuronal loss was observed in G2019S mice	G2019S mice displayed elevated levels of phosphorylated tau in the substantia nigra No abnormalities related to α-synuclein were detected	G2019S mice displayed age-dependent motor activity deficits
Daher et al. (2012)	CMV-PDGFβ-driven LRRK2 Tg mice crossed with PrP-driven α-synuclein A53T Tg mice	G2019S	DA neuron loss was not altered in G2019S/A53T bigenic mice	G2019S/A53T bigenic mice exhibited a subtle increase in α-synuclein pathology in select brainstem regions	Premature lethality was not exacerbated in G2019S/A53T bigenic mice
	LRRK2 knockout mice crossed with PrP-driven α-synuclein A53T Tg mice	LRRK2^–/–^	LRRK2 deletion did not alter DA neuron loss in A53T mice	LRRK2 deletion yields a subtle reduction in α-synuclein pathology in select brainstem regions	Behavioral phenotypes were not altered by LRRK2 deletion
Herzig et al. (2012)	Thy1-driven LRRK2 Tg mice crossed with Thy1-driven α-synuclein A53T Tg mice	G2019S, Wild-type	Not assessed	G2019S/A53T bigenic mice did not exhibit altered levels of α-synuclein protein	G2019S/A53T bigenic mice did not display exacerbated microgliosis
Bailey et al. (2013)	LRRK2 BAC Tg mice crossed with P301L tau (rTg4510) Tg mice	Wild-type	Not assessed	LRRK2/P301L-tau bigenic mice display increased accumulation and phosphorylation of insoluble tau along with enhanced cortical tau pathology	
Yue et al. (2015)	LRRK2 knockin mice	G2019S	Not observed	G2019S mice developed modest phosphorylated tau puncta and an increase in levels of phosphorylated tau protein, No differences in α-synuclein were detected	Altered DA neurotransmission
Mikhail et al. (2015)	LRRK2 BAC Tg mice crossed with P301S tau (PS19 line) Tg mice	R1441G	No exacerbation of neuronal loss was observed in R1441G/P301S-tau bigenic mice	Tau aggregation and phosphorylation is not altered in R1441G/P301S-tau bigenic mice	Motor and memory deficits are not altered in R1441G/P301S-tau bigenic mice
Xiong et al. (2017)	CamKIIα-tTA inducible LRRK2 Tg mice	G2019S, G2019S/D1994A	Not observed	G2019S mice displayed increased levels of insoluble α-synuclein protein in the cortex, striatum, and hippocampus, Tau protein was not assessed	G2019S mice exhibit amphetamine-induced behavioral deficits
Longo et al. (2017)	LRRK2 knockin mice	G2019S	Not observed	G2019S mice displayed elevated levels of phosphorylated α-synuclein protein in the striatum along with phosphorylated α-synuclein inclusions Tau protein was not assessed	G2019S mice exhibited dysfunction of DAT and VMAT2
Xiong et al. (2018)	TH-tTA inducible LRRK2 Tg mice	G2019S, G2019S/D1994A	Age-dependent DA and NE neuronal loss was observed in G2019S mice	G2019S mice exhibited elevated levels of insoluble α-synuclein protein in the striatum and ventral midbrain Tau protein was not assessed	G2019S mice displayed age-dependent motor activity deficits
Nguyen et al. (2018)	CMV-PDGFβ-driven LRRK2 Tg mice crossed with P301S tau (PS19 line) Tg mice	G2019S	Not assessed	G2019S/P301S-tau bigenic mice did not exhibit any alteration to tau protein levels, solubility, or phosphorylation state	
	LRRK2 knockout mice crossed with P301S tau (PS19 line) Tg mice	LRRK2^–/–^	Not assessed	LRRK2 deletion did not exhibit any alteration to tau protein levels, solubility, or phosphorylation state	

**BAC, bacterial artificial chromosome; DA, dopaminergic; DAT, dopamine transporter; NE, noradrenergic; Tg, transgenic; TH, tyrosine hydroxylase; tTA, Tet-transactivator; VMAT2, vesicular monoamine transporter 2.**

Subsequent genetic models sought to examine the impact of the G2019S mutation (Table 1). To date, a number of G2019S LRRK2 transgenic models have been developed, though findings have been mixed. Melrose et al. found that human G2019S LRRK2 BAC transgenic mice displayed phosphorylated tau inclusions (CP13+, 12E8+) in cortical and limbic brain regions at advanced ages (18–24 month) (Melrose et al., 2010). Human wild-type LRRK2 BAC mice were also examined in this study and found to harbor tau pathology. However, in the wild-type LRRK2 line, tau pathology was primarily restricted to the hippocampus and to a much lesser burden than in G2019S mice. Notably, neither background was found to exhibit any apparent α-synuclein pathology. Similar to the R1441C knockin model, these mice also displayed abnormal dopaminergic neurotransmission, furthering support for a common pathogenic role in modulating synaptic function (Melrose et al., 2010). While Melrose et al. found an additive effect of the G2019S mutation on tau phosphorylation, a separate study assessing a different mouse G2019S LRRK2 BAC transgenic model failed to detect any alterations (Li et al., 2010; Melrose et al., 2010). In contrast, accumulation of phosphorylated tau inclusions (PHF1+, CP13+) was not significantly elevated relative to control mice (Li et al., 2010). While a number of factors might explain this discrepancy, including variation in strain background, or transgene copy number and/or genomic integration site, it is notable that neither model exhibited neurodegeneration of midbrain dopaminergic neurons (Li et al., 2010; Melrose et al., 2010).

Despite the lack of overt neurodegeneration in these early LRRK2 mouse models, although exhibiting modest neuropathologic phenotypes, parallel efforts developed a number of additional models focused on selectively boosting transgene expression within dopaminergic neurons of the substantia nigra (Table 1). While some of these models were successful in provoking dopaminergic neurodegeneration, the impact of protein aggregation in many of these models was generally not assessed or simply not detected (Ramonet et al., 2011; Chen et al., 2012; Tsika et al., 2014; Liu et al., 2015). In one of these studies, transgenic mice expressing human G2019S LRRK2 under control of a CMV-enhanced human PDGFβ promoter were found to have an age-dependent (12–16 months) loss of dopaminergic neurons in the substantia nigra relative to controls expressing human wild-type LRRK2 from the same promoter (Chen et al., 2012). While no changes in α-synuclein or ubiquitin protein levels were observed, a nearly two-fold increase in phosphorylated tau (AT8+) was detected in the substantia nigra of G2019S LRRK2 mice (Chen et al., 2012). Unfortunately, the requirement of tau as a necessary pathologic substrate for neurodegeneration in this model was not determined. In a separate study, the G2019S mutation was expressed throughout the mouse forebrain under an inducible CamKIIα-tTA (Tet-transactivator) conditional expression system (Xiong et al., 2017). This study also examined a kinase-inactive variant, G2019S/D1994A LRRK2, under the same expression system (Xiong et al., 2017). While dopaminergic neurodegeneration was not observed with this approach, a substantial increase in the level of insoluble α-synuclein was detected in select forebrain regions of G2019S LRRK2 mice but not in G2019S/D1994A LRRK2 mice (Xiong et al., 2017). However, a similar approach examining G2019S and G2019S/D1994A LRRK2 mutations under a TH-tTA conditional expression system discovered robust age- and kinase-dependent neurodegeneration coupled with an increase in the levels of insoluble α-synuclein (Xiong et al., 2018).

In order to examine the G2019S mutation under more physiologic conditions, more recent studies have utilized G2019S LRRK2 knockin mice. While G2019S LRRK2 BAC transgenic mice previously displayed alterations in tau phosphorylation, Yue et al. (2015) found that G2019S knockin mice failed to exhibit, similarly, significant changes. However, these mice did harbor modest pathology upon examination at advanced age (18 months). Cytoplasmic accumulation of phosphorylated tau (pSer202+) was observed, including some puncta formation and neuritic pathology (Yue et al., 2015). More readily apparent were deficits in striatal dopamine release and altered mitochondrial dynamics, again supporting synaptic alterations as an early sign of LRRK2 pathogenicity (Yue et al., 2015). In a separate study, G2019S LRRK2 knockin mice were found to exhibit a two-fold increase in the level of phosphorylated α-synuclein (pSer129) in the striatum (Longo et al., 2017). This finding was coupled with histological evidence of striatal α-synuclein inclusions. Importantly, both of these findings were age-dependent (12 months) (Longo et al., 2017). In a follow-up study, primary neuronal cultures generated from G2019S knockin mice were found to exhibit elevated levels of both phosphorylated tau (AT8+) and insoluble α-synuclein (Schapansky et al., 2018). Remarkably, administration of a LRRK2 kinase inhibitor was demonstrated to reverse the effect of the G2019S mutation on altered α-synuclein solubility, though the effect of kinase inhibition on tau phosphorylation was not determined (Schapansky et al., 2018). Certain species of α-synuclein and tau are thought to synergistically interact, and experimental evidence suggest that α-synuclein and tau are able to cross seed (Lim, 2019). While the finding of alterations to both α-synuclein and tau in a LRRK2 model are intriguing, the process by which this occurs may not necessarily be linear, and subsequent studies should address this concern.

While the collective findings from LRRK2 genetic animal models have not fully clarified the impact of LRRK2 on protein aggregation, the use of LRRK2 mice in a cross breeding approach has allowed for an alternative strategy in dissecting this relationship (Table 1). One of the first *in vivo* studies examining a potential interaction between LRRK2 and α-synuclein found that crossing inducible transgenic mice lines expressing human LRRK2 (either wild-type or G2019S) and A53T α-synuclein led to an increase in the burden of α-synuclein pathology (Lin et al., 2009). The mouse lines used in this study were generated using the CamKIIα-tTA conditional expression system, with transgene expression being restricted to the forebrain. While both LRRK2 lines independently were without phenotype, crossing either line with A53T mice had an additive effect on the level of cortical α-synuclein pathology. Intriguingly, A53T mice were also crossed with *LRRK2* knockout mice and a dramatic reduction in the burden of α-synuclein pathology was observed. Taken together, these findings first suggested that LRRK2 may be involved in the progression of α-synuclein neuropathology (Lin et al., 2009). Other studies examining the interaction of LRRK2 and α-synuclein have suggested that the impact of LRRK2 on protein aggregation may be more complex. In two related studies, distinct human mutant α-synuclein transgenic mouse models with predominant hindbrain pathology were found to have no pathological interaction with LRRK2 transgenic or knockout mice (Daher et al., 2012; Herzig et al., 2012). It is possible that the discrepancy between these two studies and the former result from a regionally-restricted interaction between LRRK2 and α-synuclein, with greater potential for interaction in the forebrain where LRRK2 expression is enriched (Biskup et al., 2006; Higashi et al., 2007; West et al., 2014).

Similar to the potential interaction between LRRK2 and α-synuclein, a pathologic interplay between LRRK2 and tau has also been pursued using a cross breeding approach (Table 1). A human wild-type LRRK2 BAC transgenic mouse line crossed with inducible transgenic tau mice (expressing human P301L mutant tau under a CamKIIα-tTA conditional expression system) was shown to enhance cortical tau aggregation and increase tau phosphorylation (AT8+, CP13+, MC1+) (Bailey et al., 2013). In contrast, a human R1441G LRRK2 BAC transgenic mouse line crossed with human P301S tau mice (driven by the mouse prion promoter, PrP) found no significant interaction (Mikhail et al., 2015). While the P301S tau mice used in this study developed widespread tau pathology, no additional alterations in tau aggregation or phosphorylation were identified in these bigenic mice (Mikhail et al., 2015). Similarly, modest hippocampal neuron loss specific to the P301S tau mouse background was not exacerbated by mutant LRRK2 (Mikhail et al., 2015). As this study did not generate P301S tau/LRRK2 knockout mice, it was not clear whether tau pathology was dependent on endogenous LRRK2 activity. However, a subsequent study was able to address this question. Nguyen et al. crossed the same PrP-P301S tau mice with either LRRK2 knockout or human G2019S transgenic mice (under the CMVe-PDGFβ promoter) (Nguyen et al., 2018). Neither genetic deletion of LRRK2 nor pathogenic mutation altered tau protein levels, solubility, or phosphorylation state. In addition, histopathological assessment of these mice found no interaction between tau pathology and LRRK2 genotype (Nguyen et al., 2018).

### LRRK2 in Viral Vector-Based Models

Additional evidence supporting the involvement of LRRK2 in protein aggregation, propagation, and toxicity has come from the implementation of viral vectors in rodent models (Table 2). Importantly, the use of viral vectors offers several advantages over traditional genetic models. First, viral vector delivery offers spatiotemporal control of gene expression and avoids the potential confounding effects of developmental compensation. As well, higher levels of transgene expression are likely attainable when using this approach. In a practical sense, viral vectors may be applied across multiple genetic lines, amplifying the utility of viral vectors toward understanding disease-relevant interactions. Large-capacity viral vectors carrying full-length human LRRK2 have been used to uncover the impact of LRRK2 overexpression and pathogenic mutations on nigrostriatal pathway integrity (Table 2).

**TABLE 2:** LRRK2 in viral vector-based models.

References	Model	LRRK2 variant(s)	Neurodegeneration	Pathology/Protein aggregation	Additional findings
Lee et al. (2010)	Intrastriatal delivery of HSV vectors expressing LRRK2 in mice	G2019S, Wild-type, G2019S/D1994A	DA neuronal loss was observed in a kinase-dependent manner with greater loss observed with the G2019S vector	Not assessed	Non-selective LRRK2 kinase inhibitor treatment was demonstrated to attenuate DA neuronal loss
Dusonchet et al. (2011)	Intrastriatal delivery of Ad5 vectors expressing LRRK2 in rats	G2019S, Wild-type	DA neuronal loss was observed in rats injected with the G2019S vector	Phosphorylated tau inclusions were transiently observed in the substantia nigra α-synuclein and ubiquitin protein abnormalities were not detected	
Daher et al. (2014)	Intranigral delivery of AAV2/1 vector expressing WT α-synuclein in LRRK2 knockout rats	LRRK2^–/–^	DA neuron loss was ameliorated in LRRK2 knockout rats	LRRK2 deletion did not alter levels of α-synuclein protein	LRRK2 deletion was also demonstrated to protect against LPS-induced pathology
Tsika et al. (2015)	Intrastriatal delivery of Ad5 vectors expressing LRRK2 in rats	G2019S, G2019S/D1994N, Wild-type	Loss of TH+ fiber density in the striatum was found to be independent of LRRK2 kinase activity, DA neuron loss was not observed	Ubiquitin+ inclusions and altered neurofilament distribution were observed in the striatum Tau and α-synuclein protein abnormalities were not detected	
Daher et al. (2015)	Intranigral delivery of AAV2/1 vector expressing WT α-synuclein in LRRK2 BAC Tg rats	G2019S	DA neuron loss was shown to be exacerbated in G2019S rats	Not assessed	LRRK2 kinase inhibitor treatment was demonstrated to attenuate DA neuron loss
Andersen et al. (2018)	Intranigral delivery of AAV2/5 vector expressing A53T α-synuclein in LRRK2 knockout rats	LRRK2^–/–^	Aberrant STN burst firing activity was ameliorated in LRRK2 knockout rats	α-synuclein phosphorylation state was not altered across all conditions	STN burst firing activity was also corrected by LRRK2 kinase inhibition (PF360)
Novello et al. (2018)	Intranigral delivery of AAV2/9 vector expressing α-synuclein in LRRK2 knockin mice	G2019S	No additive effect on DA neuron loss was observed in the G2019S mice	α-synuclein pathology was found to be elevated in the substantia nigra of G2019S mice	
Nguyen et al. (2018)	Intrahippocampal delivery of AAV2/6 vector expressing either wild-type or P301S mutant tau in LRRK2 Tg mice	G2019S	Hippocampal neuron loss was not altered in G2019S mice	Hippocampal tau pathology was not altered in G2019S mice	G2019S was found to enhance the neuronal transmission of wild-type tau
	Intrahippocampal delivery of AAV2/6 vector expressing either wild-type or P301S mutant tau in LRRK2 knockout mice	LRRK2^–/–^	Hippocampal neuron loss was not altered in LRRK2 knockout mice	Hippocampal tau pathology was not altered in LRRK2 knockout mice	Endogenous LRRK2 was found to be dispensable for the neuronal transmission of wild-type tau
Kritzinger et al. (2018)	Intrastriatal delivery of HC-AdV expressing LRRK2 in mice	G2019S, D1994N	DA neuron loss was not observed, but modest striatal cell loss was observed with the G2019S vector in aged mice	No evidence of ubiquitin, tau, or α-synuclein pathology was observed	Glial LRRK2 expression was found to contribute to neuroinflammation
Mestre-Francés et al. (2018)	Intrastriatal delivery of CAV-2 vectors expressing LRRK2 in *M. murinus* (non-human primate)	G2019S	DA neuron loss was observed with both G2019S and GFP control vectors	Phosphorylated tau and α-synuclein pathology were observed with the G2019S vector	
Nguyen et al. (2019)	Intrastriatal delivery of Ad5 vectors expressing LRRK2 in rats	G2019S, Wild-type, G2019S/K1906M	DA neuron loss was observed in a kinase-dependent manner with greater loss observed with the G2019S vector	Phosphorylated tau inclusions were observed in the substantia nigra across all vectors, APP+ inclusions and degenerative neuritic changes in the striatum were observed with the G2019S vector Changes to α-synuclein protein were not detected	LRRK2 kinase inhibitor (PF360) treatment was demonstrated to attenuate DA neuron loss
di Caudo et al. (2020)	Intranigral delivery of CAV-2 vectors expressing LRRK2 in *M. fascicularis* (non-human primate)	G2019S	DA neuron loss was observed in both G2019S and GFP control vectors	Phosphorylated tau pathology was observed with both the G2019S and GFP control vectors with a modest increase in select regions for the G2019S vector α-synuclein protein was not assessed	

**AAV, adeno-associated virus; Ad5, human adenovirus serotype 5; BAC, bacterial artificial chromosome; CAV-2, canine adenovirus serotype 2; DA, dopaminergic; HSV, herpes simplex virus; LPS, lipopolysaccharide; Tg, transgenic; WT, wild-type.**

In a herpes simplex virus (HSV) vector mouse model, either human wild-type, G2019S, or G2019S/D1994A LRRK2 variants (kinase-inactive) were delivered via intrastriatal injection (Lee et al., 2010). Using this approach, modest dopaminergic neuronal loss was observed with the wild-type variant with significantly greater loss with the G2019S variant. Concurrently, the kinase-inactive variant did not induce any observable loss and concurrent administration of early yet non-selective LRRK2 kinase inhibitors was shown to attenuate neurodegeneration in this model. Accordingly, these findings support the importance of functional kinase activity in mediating dopaminergic neurodegeneration. Unfortunately, protein aggregation was not assessed in this study so it is not clear how tau or α-synuclein might be involved with LRRK2 delivered via HSV vectors.

Another study examining human LRRK2 utilized human adenoviral vectors (Ad5) driven by a neuronal-specific synapsin-1 promoter and found similar findings to the HSV model (Dusonchet et al., 2011). In this approach, intrastriatal delivery of Ad5 carrying G2019S LRRK2 induced robust neurodegeneration in the substantia nigra of adult rats, while no effect was observed with the wild-type LRRK2 or GFP vectors. Intriguingly, transient induction of neuritic inclusions was observed in the substantia nigra with both wild-type and G2019S vectors. These inclusions exhibited positive immunostaining for phosphorylated tau (AT8+) but not for α-synuclein (Dusonchet et al., 2011). Following these initial findings, a second study examined the impact of the Ad5-LRRK2 on striatal pathology (Tsika et al., 2015). Intraneuronal ubiquitin-positive inclusions were observed in the striatum of rats injected with the G2019S LRRK2 vector, but not with a G2019S/D1994N vector. As well, degenerative neuritic changes and altered phosphorylated neurofilament distribution were observed in the striatum in a kinase-dependent fashion (Tsika et al., 2015). Unfortunately, the G2019S/D1994N mutation was shown to alter LRRK2 protein stability, partly confounding the validity of this synthetic inactive mutation in determining kinase-dependency. However, an additional study performing intrastriatal injection of Ad5-LRRK2 vectors at a higher viral titer in rats utilized an alternative stable kinase-inactivating mutation, G2019S/K1906M (Nguyen et al., 2019). In this study, phosphorylated tau inclusions were detected in the substantia nigra across all LRRK2 variants, suggesting that the effect is not specific to the G2019S mutation or kinase activity (Nguyen et al., 2019). Concurrently, biochemical analysis determined that tau protein levels, phosphorylation state, and solubility were not altered in response to Ad5-LRRK2 vectors (Nguyen et al., 2019). Additionally, ubiquitin-positive inclusions in the striatum did not appear to be dependent on kinase activity and none of the LRRK2 vectors induced detectable α-synuclein pathology. However, in this study, the G2019S LRRK2 vector alone induced APP-positive axonal inclusions and degenerative neuritic changes in the striatum (Nguyen et al., 2019). In a separate study, high-capacity adenoviral vectors (HC-AdV) expressing G2019S or G2019S/D1994N LRRK2 variants from a ubiquitous CAG promoter were delivered to the striatum of mice (Kritzinger et al., 2018). In this instance, modest age-dependent neuroinflammation and vacuolization of striatal white fiber tracts was induced by G2019S LRRK2 only in old animals, but protein aggregation was not detected (Kritzinger et al., 2018). Outside of rodents, a helper-dependent canine adenovirus type 2 (CAV-2) vector expressing human wild-type or G2019S LRRK2 was utilized in a non-human primate model, *Microcebus murinus* (Mestre-Francés et al., 2018). In this model, nigral neurodegeneration was equivalently observed with both LRRK2 variants and with a GFP control, making interpretation of this LRRK2 model challenging, although an increase in α-synuclein and tau phosphorylation (pSer129-αSyn+ and pSer396-tau+, respectively) was identified due to the G2019S variant (Mestre-Francés et al., 2018). An additional study assessing CAV-2-LRRK2 vectors in macaques, similarly, found an elevation of phosphorylated tau (PHF+) immunostaining in the substantia nigra of G2019S LRRK2-injected animals (di Caudo et al., 2020).

In contrast to viral vectors delivering LRRK2, viral vectors have also been used to introduce α-synuclein overexpression in neuronal populations of interest. Moreover, these vectors have proven to reliably induce neurodegeneration indicative of α-synuclein-dependent toxicity (Ulusoy et al., 2010). In order to investigate the contribution of LRRK2 toward protein aggregation and neuropathology, α-synuclein viral vectors have been introduced into LRRK2 rodent models in an approach that mimics the generation of bigenic models discussed previously. Toward this, an AAV2/1 vector delivering human wild-type α-synuclein to the substantia nigra of adult rats was shown to induce ∼30% loss of dopaminergic neurons (Daher et al., 2014). Remarkably, when the same vector was introduced in *LRRK2* knockout rats, the neurodegenerative phenotype was ameliorated. While it was difficult to assess the extent of α-synuclein pathology in this model due to neuronal loss, the level of α-synuclein pathology was shown to inversely correlate with cell loss (Daher et al., 2014). In a subsequent study, AAV2/1-α-synuclein was delivered to the substantia nigra of G2019S LRRK2 BAC transgenic rats (Daher et al., 2015). Here, dopaminergic cell loss was determined to be significantly increased in the G2019S LRRK2 rats relative to control animals, and importantly, this effect was found to be kinase-dependent as pharmacologic kinase inhibition reversed the effect (Daher et al., 2015).

Use of an AAV2/5-α-synuclein vector delivered to the substantia nigra of rats was shown to induce aberrant neuronal firing activity in the subthalamic nucleus (Andersen et al., 2018). Intriguingly, this pathologic effect was not present in *LRRK2* knockout rats and was reversed in wild-type rats following administration of a LRRK2 kinase inhibitor (Andersen et al., 2018). However, a later study found that long-term administration of a LRRK2 kinase inhibitor failed to protect against this abnormal α-synuclein-mediated neuronal dysfunction in rats (Andersen et al., 2019). Another study examined the impact of the G2019S mutation on virally mediated α-synuclein pathology using a more physiologically relevant model. Using G2019S LRRK2 knockin mice, along with wild-type controls, Novello et al. (2018) found that stereotactic injection of AAV2/9 vectors expressing human A53T α-synuclein was sufficient to induce ∼50% loss of dopaminergic neurons in the substantia nigra. However, no additive neurodegenerative effect was conferred by the G2019S mutation in the knockin model. Rather, G2019S knockin mice were found to exhibit a nearly two-fold increase in the level of nigral α-synuclein pathology (as measured by pSer129+ immunostaining) (Novello et al., 2018). This study also sufficiently characterized the observed α-synuclein pathology as resistant to proteinase-K treatment, indicative of fibrillar aggregates (Novello et al., 2018). While these findings suggest that LRRK2 may, in part, mediate the neurodegenerative phenotype of α-synuclein, a separate study identified LRRK2 activation via proximity ligation assays in the substantia nigra of rats following intranigral injection of AAV2/1 vector expressing human wild-type α-synuclein (Maio et al., 2018). Thus, while overexpression of LRRK2 via viral vector delivery is not generally sufficient to induce α-synuclein pathology in the face of neurodegeneration, the reverse seems to be true in that virally-mediated α-synuclein pathology may be potentiated by LRRK2 activity.

While less is known about the function of LRRK2 activity on virally-induced tau pathology, alterations to tau biology have been a common observation in LRRK2 animal models. The generation of bigenic mice examining the impact of LRRK2 genotype in mutant tau transgenic mice model previously failed to detect substantial changes to tau (Nguyen et al., 2018). However, additional experimentation suggests that LRRK2 genotype may alter other aspects of virally-mediated tau pathology. Utilizing AAV2/6 vectors to deliver human P301S tau via intrahippocampal injection, Nguyen et al. compared LRRK2 knockout and G2019S LRRK2 transgenic mice (Nguyen et al., 2018). While P301S tau induced ample tau pathology in the hippocampus, LRRK2 genotype was not determined to alter the burden of tau pathology or hippocampal neuronal loss (Nguyen et al., 2018). In contrast, use of an AAV2/6 vector co-expressing human wild-type tau and a GFP reporter revealed an impact of LRRK2 genotype on the neuronal transmission of tau within defined hippocampal circuits (Nguyen et al., 2018). While endogenous LRRK2 was found to be dispensable for tau transmission, a two-fold increase in transmission to both ipsilateral and contralateral CA3 regions was observed in G2019S LRRK2 transgenic mice (Nguyen et al., 2018). Notably, this experiment found no evidence of tau aggregation or neurodegeneration following viral vector delivery of wild-type tau, suggesting that LRRK2 activity may instead serve to accelerate the spread of tau pathology from cell-to-cell within defined circuits.

### LRRK2 in Protein-Based Models

Accumulating evidence from human and experimental animal studies supports the pathological transmission of α-synuclein and tau species in PD and other neurodegenerative diseases (Frost and Diamond, 2009). Notably, both α-synuclein and tau pathology have been observed in fetal neural grafts of PD patients at autopsy (Kordower et al., 2008; Li et al., 2008, 2016; Cisbani et al., 2017). While the mechanistic explanations surrounding the stereotypical spatiotemporal spread of tau and α-synuclein pathology remains uncertain, it is clear that protein aggregation is inducible across interconnected neuronal populations and in a sequential pattern. Experimental models have demonstrated the cell-to-cell release and uptake of toxic protein species (Volpicelli-Daley et al., 2011, 2016; Mao et al., 2016). Whether such direct transmission models are relevant to human disease still remains to be determined but understanding the progression of inclusion formation within the context of *in vivo* models is certain to be invaluable. The development of α-synuclein pre-formed fibrils (PFF) as an experimental tool has allowed for the examination of cell-to-cell transmission of α-synuclein and resulting induction of pathology (Luk et al., 2009; Volpicelli-Daley et al., 2011; Duffy et al., 2018; Polinski et al., 2018; Patterson et al., 2019). Importantly, PFF-induced α-synuclein inclusions recapitulate key features of human Lewy pathology. Direct delivery of α-synuclein PFFs into the rodent brain has provided a robust model in which to dissect mechanisms of protein misfolding, aggregation, and spread *in vivo*. While the advancement and implementation of tau PFFs *in vivo* have been more limited, both α-synuclein and tau have been used to further a mechanistic understanding of pathological aggregation in the mammalian brain.

The introduction of α-synuclein PFFs into LRRK2 rodent models has provided a robust system in which to determine how LRRK2 activity relates to the progression and propagation of α-synuclein aggregation as well as the impact of pathogenic LRRK2 activity on α-synuclein mediated neurodegeneration (Table 3). Importantly, inoculation with α-synuclein PFFs and subsequent induction of Lewy-like pathology has been demonstrated to be dependent on the presence of endogenous α-synuclein (Volpicelli-Daley et al., 2011; Luk et al., 2012). Moreover, the level of pathologic burden across brain regions appears related to the regional variation in α-synuclein expression, such that brain regions associated with elevated levels of α-synuclein are associated with a greater severity of α-synuclein pathology while regions with lower levels of expression are associated with relative resistance to pathology (Henderson et al., 2019a). While the PFF model is less useful in determining the contribution of LRRK2 mutations toward the direct initiation of α-synuclein or tau pathology, other aspects of this model have proven instrumental. Notably, the PFF model has robustly demonstrated the importance of neural connectivity rather than area locality for the transmission of pathology, further supporting a cell-to-cell transmission mechanism in the progression of pathology (Henderson et al., 2019a). This feature is readily observed in the established rodent intrastriatal injection paradigm, in which the induction of α-synuclein pathology progressively accumulates in neuronal populations innervating the striatum. This observation is readily apparent in the substantia nigra, where progressive α-synuclein pathology peaks at 2–3 months following injection followed by modest neurodegeneration by 6 months (Luk et al., 2012; Paumier et al., 2015). The PFF model thus allows for examination of the spatiotemporal spread of protein substrates coupled with insight into selective vulnerability of affected regions.

**TABLE 3:** LRRK2 in protein-based models.

References	Model	LRRK2 variant(s)	Neurodegeneration	Pathology/Protein aggregation	Additional findings
Volpicelli-Daley et al. (2016)	Intranigral delivery of PFF in LRRK2 BAC Tg rat	G2019S	Not observed	G2019S rats exhibited increased α-synuclein inclusion burden in the substantia nigra	
Zhao et al. (2017)	Intrastriatal delivery of PFF in mice treated with LRRK2 ASO	Not Applicable	Administration of LRRK2 ASO reduced PFF-induced DA neuron loss	Administration of LRRK2 ASO reduced PFF-induced α-synuclein pathology	
Henderson et al. (2019a)	Intrastriatal delivery of PFF in LRRK2 BAC Tg mice	G2019S	G2019S mice exhibited a modest increase in DA neuronal loss	G2019S mice displayed elevated α-synuclein pathology in select brain regions	G2019S mice demonstrated altered dynamics of pathological α-synuclein spread
Henderson et al. (2019b)	Intrastriatal delivery of PFF in mice treated with LRRK2 kinase inhibitor (MLi-2)	Not Applicable	MLi-2 administration did not alter PFF-induced DA neuronal loss	MLi-2 administration did not alter PFF-induced α-synuclein pathology	MLi-2 administration did not impact the PFF-induced behavioral profile
Bieri et al. (2019)	Intrastriatal delivery of PFF in LRRK2 BAC Tg mice	G2019S	G2019S mice demonstrated enhanced DA neuronal loss	G2019S mice displayed an increased α-synuclein inclusion burden in the substantia nigra	G2019S mice injected with PFF exhibited motor activity deficits and an altered neuroinflammatory profile

**ASO, antisense oligonucleotide; BAC, bacterial artificial chromosome; DA, dopaminergic; PFF, preformed fibrils of α-synuclein; Tg, transgenic.**

The implementation of the PFF model in comparative genetic mouse models has facilitated a greater understanding of how LRRK2 activity might alter the progression of pathology at the connectome level (Table 3). In a recent study by Henderson et al., regional variation in the burden of α-synuclein pathology was observed in G2019S LRRK2 BAC transgenic mice compared to non-transgenic controls (following intrastriatal PFF injection), such that G2019S mice displayed both higher and lower levels of α-synuclein pathology across distinct brain regions (Henderson et al., 2019a). Intriguingly, a significant increase in the burden of α-synuclein pathology was observed in the cortex of G2019S mice. Robust LRRK2 transgene expression is present in the cortex of G2019S mice, suggesting that heightened LRRK2 levels may potentiate α-synuclein pathology in this region. Indeed, G2019S mice were shown to harbor a greater burden of pathology in regions that were resilient in non-transgenic mice. In terms of dopaminergic neurodegeneration, G2019S mice displayed a modest increase in nigral neuronal loss which was reflective of elevated α-synuclein pathology in the substantia nigra. Additional studies have sought to identify whether LRRK2 is required for α-synuclein transmission and neurodegeneration in the PFF model. Administration of MLi-2, a selective LRRK2 kinase inhibitor, in mice inoculated with α-synuclein PFFs demonstrated no significant impact on the induction of α-synuclein pathology in several regions of interest or alterations in dopaminergic cell loss in the substantia nigra (Henderson et al., 2019b). While these findings suggest an additive, but not critical, role for mutant LRRK2 in promoting α-synuclein pathology *in vivo*, other studies have produced contrasting results. Toward this, administration of antisense oligonucleotides directed against LRRK2 were found to reduce α-synuclein inclusion burden and neurodegeneration in an α-synuclein PFF model (Zhao et al., 2017). As well, primary neuronal cultures derived from either LRRK2 knockin or knockout mice were found to exhibit differential levels of α-synuclein aggregate pathology following PFF administration, with LRRK2 knockout playing a protective role (MacIsaac et al., 2020).

Despite the mixed findings, several additional studies have noted either an acceleration or exacerbation of PFF-induced α-synuclein pathology in the context of the pathogenic G2019S mutation. While enhanced LRRK2 activity does not appear to enhance neuronal uptake of fibrils *in vitro*, it is possible that mutant LRRK2 impacts diverse biological processes that result in the potentiation of aggregation. Volpicelli-Daley et al. found that overexpression of G2019S LRRK2 in a BAC transgenic rat model increased the number of α-synuclein and ubiquitin-positive inclusions in the substantia nigra at 1 month following unilateral intranigral PFF injection (Volpicelli-Daley et al., 2016). Likewise, Bieri et al. (2019) found that bilateral intrastriatal injection of α-synuclein PFF into G2019S LRRK2 BAC mice led to an exacerbation of phosphorylated α-synuclein aggregates across interconnected brain regions including the substantia nigra. While the effect of the G2019S mutation on protein aggregation was significant at 1, 3, and 6 months following PFF injection, the effect size was diminished over time. This observation supports that PFF-induced pathology may be subject to a thresholding effect, and, additionally, that LRRK2 may have both an immediate impact on the induction of pathology as well as longitudinal control of aggregate formation. Of interest, dopaminergic cell loss was more severe in the G2019S background, which could be resultant from the increased burden of aggregate pathology or from an intrinsic impact of G2019S LRRK2 on neuronal survival (Bieri et al., 2019). In total, LRRK2 appears to subtlety modulate the burden of protein aggregate pathology (Table 3), though the mechanism by which this occurs remains to be fully uncovered.

## Conclusion

Given the heterogenous pathological landscape of *LRRK2*-PD, it remains uncertain whether and how LRRK2 activity might impact the development of protein aggregate pathology. Although there is evidence for a potential interaction between LRRK2 and α-synuclein, or LRRK2 and tau, many studies in animal models have had conflicting results. This likely arises from variations in experimental approach and models, and further lack of understanding of LRRK2 biology. Even when an interaction is identified, the mechanisms by which LRRK2 induces, promotes, or accelerates aggregation or neurodegeneration are not well-defined. While some studies suggest that LRRK2 is not critical for the development of α-synuclein or tau pathology, the opposite paradigm still remains to be fully explored. Whether α-synuclein or tau are essential for mutant LRRK2-induced neuropathology has not been formally evaluated *in vivo*. Subsequent studies will clarify this interaction and seek to place the significance and interaction of LRRK2, α-synuclein, and tau within PD-associated neurodegenerative processes. Toward this aim, LRRK2 animal models have been critical in developing our current understanding of LRRK2 pathobiology and, along with technological refinements and advances in this area, they are poised to play a significant role in future mechanistic studies of LRRK2, including protein aggregation pathways.

## Author Contributions

DD: writing – original draft. DM: writing – reviewing and editing. Both authors contributed to the article and approved the submitted version.

## Conflict of Interest

The authors declare that the research was conducted in the absence of any commercial or financial relationships that could be construed as a potential conflict of interest.
